# Revisiting Virchow’s triad: exploring the cellular and molecular alterations in cerebral venous congestion

**DOI:** 10.1186/s13578-024-01314-5

**Published:** 2024-10-23

**Authors:** Chen Zhou, Yifan Zhou, Wei Ma, Lu Liu, Weiyue Zhang, Hui Li, Chuanjie Wu, Jian Chen, Di Wu, Huimin Jiang, Xunming Ji

**Affiliations:** 1 Beijing Institute of Brain Disorders, Laboratory of Brain Disorders, Ministry of Science and Technology, Collaborative Innovation Center for Brain Disorders, Beijing Advanced Innovation Center for Big Data-Based Precision Medicine, Capital Medical University, Beijing, 100069 China; 2 Department of Neurology, Xuanwu Hospital, Capital Medical University, Beijing, 100053 China; 3 Beijing Advanced Innovation Center for Big Data-Based Precision Medicine, School of Biological Science and Medical Engineering, Beihang University, Beijing, 100191 China; 4 Department of Neurosurgery, Xuanwu Hospital, Capital Medical University, Beijing, 100053 China

**Keywords:** Stroke, CVT, Virchow’s triad, Cerebral venous congestion, Endothelial injury

## Abstract

**Background:**

Cerebral venous thrombosis (CVT) is a rare but serious condition that can lead to significant morbidity and mortality. Virchow’s triad elucidates the role of blood hypercoagulability, blood flow dynamics, and endothelial damage in the pathogenesis of CVT. Cerebral venous congestion (CVC) increases the risk of cerebral venous sinus thrombosis and can lead to recurrent episodes and residual symptoms. However, the precise mechanism by which blood congestion leads to thrombosis remains unclear. Our objective was to investigate the cellular and molecular alterations linked to CVC through analysis of the pathological morphology of venous sinus endothelial cells and transcriptomic profiling.

**Results:**

This study demonstrated a remarkable correlation between CVC and the phenotypic transformation of endothelial cells from an anticoagulant to a procoagulant state. The findings revealed that cerebral venous stasis results in tortuous dilatation of the venous sinuses, with slow blood flow and elevated pressure in the sinuses and damaged endothelial cells of the retroglenoid and internal jugular vein ligation (JVL) rat model. Mechanistically, analysis of transcriptomic results of cerebral venous sinus endothelial cells showed significant activation of platelet activation, complement and coagulation cascades pathway in the JVL rats. Furthermore, the expression of von Willebrand factor (vWF) and coagulation factor VIII (F8) in the complement and coagulation cascades and Fgg and F2 in the platelet activation was increased in the cerebral venous sinuses of JVL rats than in sham rats, suggesting that endothelial cell injury in the venous sinus induced by CVC has a prothrombotic effect. In addition, endothelial cell damage accelerates coagulation and promotes platelet activation. Significantly, the concentrations of vWF, F2 and F8 in venous sinus blood of patients with internal jugular vein stenosis were higher than in their peripheral blood.

**Conclusion:**

Collectively, our data suggest that CVC can induce endothelial cell damage, which then exhibits a procoagulant phenotype and ultimately increases the risk of CVT. This research contributes to our understanding of the pathophysiology of CVC associated with procoagulant factors and reexamines the components of Virchow’s triad in the context of CVC.

**Supplementary Information:**

The online version contains supplementary material available at 10.1186/s13578-024-01314-5.

## Introduction

CVT is a special type of venous thrombosis caused by thrombosis of the dural sinus, cortical vein, deep vein and other intracerebral venous systems from various causes and is a potentially life-threatening disease that accounts for approximately 0.5–1.0% of all cerebrovascular diseases [1–4].

The etiology and risk factors for CVT are complex; more than 150 years ago, Rudolf Virchow proposed a triad of events required for thrombus formation, including abnormal changes in the vessel wall, blood flow, and blood constituents [5]. It is believed that multiple causes of blood hypercoagulation, hemodynamic abnormalities, endothelial damage, or vessel wall damage can lead to the development or recurrence of thrombosis [5, 6]. The established etiologies of CVT encompass a spectrum of conditions associated with systemic venous thrombosis, particularly various hypercoagulable states that typically manifest prior to the onset of CVT. These conditions include adenocarcinoma, polycythemia vera, thrombocythemia, leukemia, sickle cell disease, pregnancy and the postpartum period. Additionally, other factors, such as direct cranial trauma, neurosurgical interventions involving a venous sinus, and bacterial meningitis, are also identifiable precursors to CVT [7, 8].

CVC refers to a clinical condition characterized by decreased venous outflow from the brain. This phenomenon is frequently associated with conditions such as long periods of weightlessness [9], venous sinus or vein occlusion/stenosis of the head and neck [10], and chronic heart failure (termed “backward failure”), which raises jugular venous pressure that is transmitted to the cerebral circulation [11, 12]. A recent study of 11 International Space Station crew members revealed that six exhibited stagnant or retrograde flow in the internal jugular vein around flight day 50, and one crew member experienced the development of an occlusive internal jugular vein thrombus during spaceflight, which highlights a previously unidentified risk associated with CVC in space [9]. However, limited research has been conducted on the variables linked to the likelihood of venous thrombosis following CVC, which is caused by the stenosis of venous blood flow. A study involving 92 patients with CVT and 107 patients with internal jugular vein stenosis demonstrated that nonthrombotic internal jugular venous stenosis may be a potential risk factor for CVT [13]. A prior study conducted by our team demonstrated that the narrowing internal jugular vein (IJV), as detected via color Doppler flow imaging (CDFI), may disturb intracranial venous hemodynamics, thereby promoting CVT [14]. Cerebral venous stasis has been found to lead to increased pressure in the cerebral venous sinuses, tortuous and dilated vascular patterns or venous plexus, and the formation of CVC [15, 16]. In addition, CVC may also be associated with cognitive impairment [12, 17], degenerative disease [18, 19], brain fog [20], transient global amnesia [14], and tinnitus [21]. Importantly, venous sinus occlusion/stenosis is also a risk factor for cerebral venous sinus thrombosis and is a trigger for recurrent venous sinus thrombosis and residual symptoms [14, 22]. Significantly, studies conducted on animal models of cerebral venous sinus thrombosis have shown that the injection of thrombin into the venous sinus rarely results in thrombus formation in the venous sinus with normal venous sinus flow, whereas when the proximal end of the venous sinus is ligated or semitruncated (which results in congestion in the venous sinus) and followed by the injection of thrombin, thrombus formation occurs within the venous sinus [23]. This effect indicates that CVC plays a crucial role in the formation of thrombi. However, previous studies have emphasized the hemodynamic impacts of CVC and ignored the molecular changes in vascular endothelial pathological remodeling after venous congestion. We found that CVC caused by cerebral venous occlusion, in addition to the morphological changes of venous sinus hypertension (such as venous sinus tortuosity and dilation), causes damage to the venous sinuses and even the cerebral vascular endothelium, which leads to enhanced endothelial platelet activation and a procoagulant phenotype. The findings of this study elucidate the molecular pathology underlying cerebral venous stasis and its association with an elevated risk of venous thrombosis or thrombus recurrence in CVT, as well as cerebral venous congestion in space environments. Our research revisits and updates the understanding of the Virchow’s triad in the context of CVT and thrombophilias during space travel, identifying specific molecular alterations related to platelet activation and the complement and coagulation cascades, offering valuable insights and potential targets for the prevention of these conditions. Furthermore, the results affirm the necessity and appropriateness of current clinical practice of administering anticoagulation therapy for 6–12 months following CVT to prevent thrombotic recurrence.

## Materials and methods

### Animals and human samples

All rat experiments were performed in accordance with the guidelines of the Capital Medical University and approved by the Animal Care and Use Committee. Adult Sprague Dawley (SD) rats were obtained from Vital River (Beijing, China). The JVL rat model was performed as previously described [24].

All patients were recruited from Xuanwu Hospital, Capital Medical University (Beijing, China). Patients experiencing CVC who were enrolled in this study between January 2024 and March 2024 were consecutively included in this study. We collected plasma from superior sagittal sinus (SSS) and elbow vein of patients with CVC that were diagnosed by MRI + MRV, CT + CTV, or DSA; aged ≥ 16 years. All participants provided written informed consent before information collection and abided by the ethical principles described in the current revision of the Declaration of Helsinki, and the study was approved by the ethics committee of Xuanwu Hospital, Capital Medical University (KS2024016).

### Measurement of internal pressure in the SSS

To facilitate monitoring of SSS pressure, five SD rats (of both genders, weighing 250–300 g) were anesthetized using ketamine/xylazine and maintained at a body temperature of 37 °C using a heating pad. A stereotaxic apparatus was utilized to stabilize the rat’s head and create a 2 mm*3 mm notch in the midline of the parietal bone of the skull with a cranial drill to expose the SSS. Subsequently, the detector microcatheter of the Cranial Pressure Monitoring Instrument (FOP-LS-PT9-11, FISO) was inserted into the SSS of the rat via a pre-plasticized glass capillary tube. The rats were held in a stable position while pressure readings were recorded for 100 s, with pressure values recorded once per second. The average pressure value at each time point was then calculated and the differences between the groups were compared.

### Quantitative real-time PCR

Total RNA was isolated with Trizol (Invitrogen) and 1 µg was reverse transcribed with cDNA Synthesis kit (Qiagen). Quantitative real-time PCR was performed with SYBR Green SuperMix. The expression levels were normalized to GAPDH. Primer sequences are listed in Supplementary Table 1.

### Isolation of cerebral microvessels

Microvessels were isolated from the brains of rats using methods from Lee et al. [25]. The meninges were removed from the cerebral cortical. Separate out the cerebral cortex and homogenized in ice-cold DPBS using a Dounce homogenizer. The homogenate was centrifuged (4 °C) at 2000 g for 5 min, and then resuspended with 15% (wt/vol) 70-kDa dextran and centrifuged (4 °C) at 10,000 g for 15 min. Microvessels were collected with 40-µm cell strainer for ELISA or immunofluorescence.

### vWF, F2, F8 and P-selectin ELISA

The concentrations of the proteins vWF, F2, F8 and P-selectin were measured in cerebral venous sinuses from sham or JVL rats, using standard commercially available ELISA kits according to the manufacturer’s instructions (E-EL-R1079c for vWF; Elabscience, China. SEA820Ra for F2, SEB878Ra for F8, SEA569Ra for P-selectin; CLOUD-CLONE CROP, Wuhan, China). Briefly, the cerebral venous sinuses of sham and JVL rats were homogenized, and then centrifuged for 15 min at 4℃ and 12,000 g, the supernatant was subsequently processed for ELISA. The concentrations of vWF, F2, and P-selectin in cerebral venous sinuses are standardized by protein quantification. The serum levels of vWF, F2 and F8 in human were measured according to the instructions provided in the ELISA kit (SEA833Hu for vWF, SEA820Hu for F2, SEB878Hu for F8; CLOUD-CLONE CROP, Wuhan, China).

### Coagulation/blood assays/ measurement of prothrombin time

Blood samples were collected from SSS into 3.2% (w/v) sodium citrate. Plasma was prepared by centrifuging at 4500 g for 15 min. The following assays were performed to measure coagulation and fibrinolysis states.

In the prothrombin time (PT) assay, 45 µL plasma was mixed with 5 µL polysaccharide, then 100 µL of TT reagent was added, and record the prothrombin time using a coagulometer (STart^®^ analyzer).

For activated partial thromboplastin time (APTT) assay, a total of 50 µL of APTT reagent and 45 µL plasma mixed with 5 µL polysaccharide and was added to the cuvette; after incubation of the mixture for 3 min, 100 µL of CaCl_2_ (0.025 M) were added. Then, APTT was recorded.

For thrombin time (TT) assay, 45 µL plasma was mixed with 5 µL polysaccharide, and the clotting time was recorded after addition of 50 µL TT reagent.

The determination of fibrinogen concentration was conducted with 45 µL plasma and 5 µL polysaccharide. After incubating at 37 °C, add 100 µL multifibren reagent and record the fibrinogen values.

### Immunofluorescence

Microvessels or meninges were rinsed in TBST and blocked with 5% goat serum for 1 h at RT and incubated with primary antibodies overnight at 4℃. Samples were then washed in TBST and incubated with appropriate secondary antibodies for 1 h at RT. Nuclei were stained with DAPI (Sigma) for 5 min at RT. Images were acquired with a LSM880 confocal microscope (ZEISS) and analyzed with ImageJ. The antibodies used in the experiment are shown in Supplementary Table 2.

### Platelets isolation

Rats were anaesthetized with ketamine/xylazine. Blood from rats was collected from cardiac or SSS in a tube with 3.2% sodium citrate. Subsequently, blood was centrifuged at 250 g for 5 min and platelet rich plasma (PRP) was isolated. PRP was further centrifuged at 600 g for 10 min and the platelet pellet was suspended and washed twice with Tyrode’s buffer (20 mM HEPES, 134 mM NaCl, 0.34 mM Na_2_HPO_4_, 2.9 mM KCl, 12 mM NaHCO_3_, 5 mM glucose, 0.35% bovine serum albumin (pH 7.0)) containing apyrase 0.02 U/mL and prostacyclin (PGI2) 0.5 µM to prevent platelet activation.

### Flow cytometry

For platelet activation, diluted platelet suspension was divided into 100 µl each tube, stimulated with 10 µl PBS or thrombin for 10 min, an PE-conjugated P-selectin antibody was stained for detecting platelet activation, and fixed with 100 µl 2% formalin. Fluorescence intensity was measured on flow cytometer (BD). For sorting of venous sinus endothelial cells, the cerebral venous sinuses of 6 sham rats and 6 JVL rats were digested with collagenase, and then CD45^−^CD31^+^ endothelial cells were sorted out by flow cytometry for transcriptome sequencing.

### ATP release

ATP release from the supernatant after thrombin stimulation of platelets was measured using an ATP Bioluminescent Assay Kit (Beyotime) according to manufacturer’s instructions.

### Transmission electron microscopy (TEM)

SSS were quickly dissected and fixed in electron microscopy fixative for 72 h at 4 °C. Afterwards, the samples were transferred to PBS and sectioned in a sagittal manner at 1 mm intervals. Samples were then fixed in 1% osmium tetroxide and dehydrated through a series of graded ethanol and embedded in Epon 812 resin. Images were recorded by transmission electron microscope.

### RNA-seq

RNA-seq transcriptome library was prepared following ABI StepOnePlus Real-Time PCR System (Thermo Fisher Scientific, MA, USA) using 1 mg of total RNA. Then the mRNA was reverse transcribed into cDNA, purified and amplified by PCR to obtain the final library. Finally, the paired-end RNA-seq sequencing library was sequenced with the DNBSEQ (SE50). R statistical package software DEseq2 (http://www.bioconductor.org/packages/stats/bioc/edgeR/, version 3.14.0) was utilized for differential expression analysis. In addition, GO annotation analysis and KEGG function enrichment analysis (*P*-adjust ≤ 0.05) are performed by Clusterprofiler (version 4.6.0).

### Statistical analysis

Statistical analysis was performed with GraphPad Prism 7. Unpaired Student’s t-tests were used to assess statistical significance. For all experiments, each test was two-sided and *P* < 0.05 was considered statistically significant.

## Results

### Cerebral venous stasis contributes to cerebral venous congestion and endothelial cell damage

**Fig. 1:**
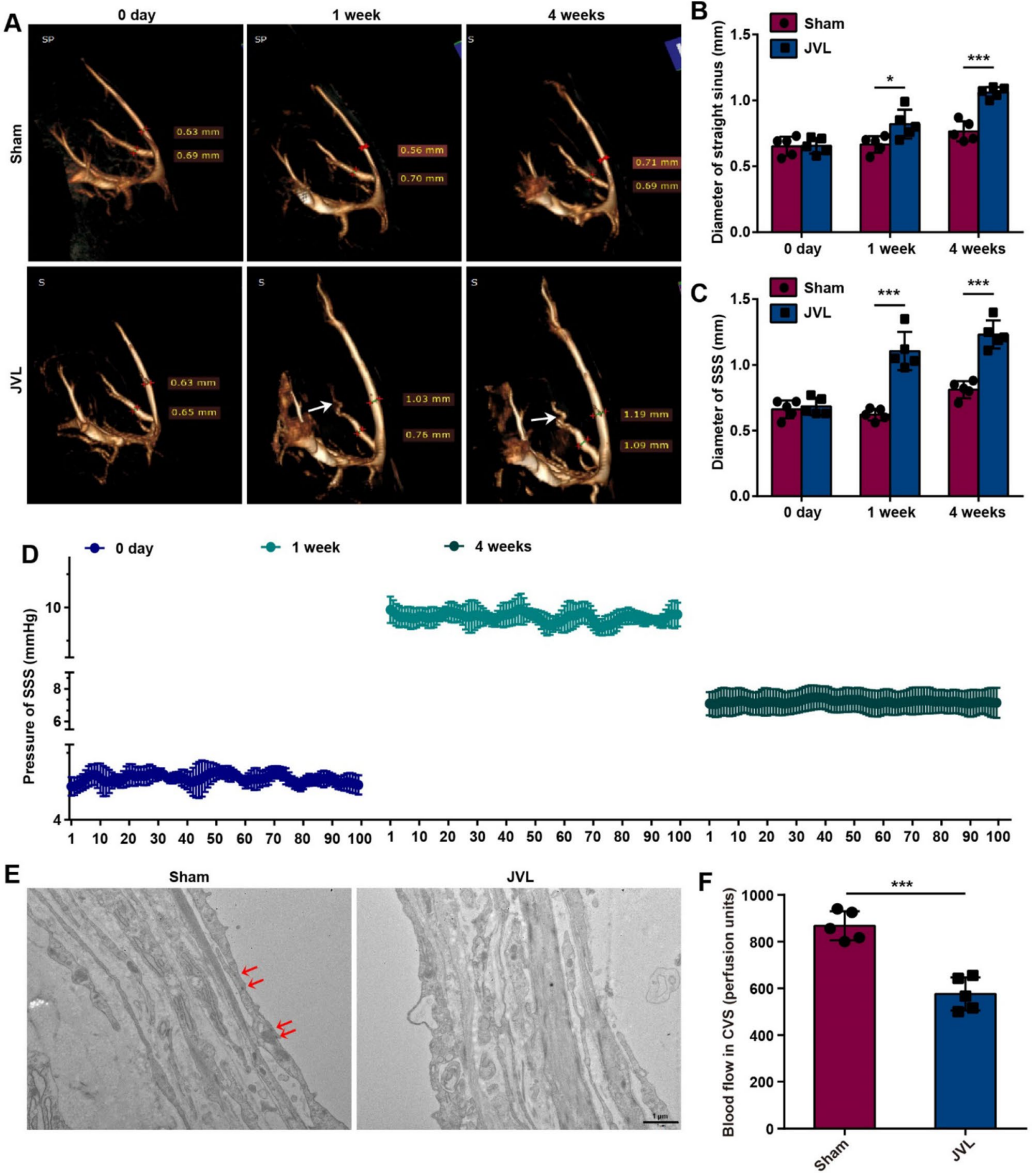
Cerebral venous stasis results in tortuosity and dilatation of the venous sinus, endothelial cell damage, and slow blood flow. (**A**) MRV of the cerebral venous sinuses of sham and JVL rats at 0 days, 1 week and 4 weeks, the white arrows indicate tortuous and dilated straight sinus (*n* = 5, 2 females and 3 males). (**B**-**C**) The diameters of the straight sinus (**B**) and SSS (**C**) in sham and JVL rats at 0 days, 1 week and 4 weeks (*n* = 5, 2 females and 3 males). (**D**) Pressure in the SSS of JVL rats before surgery and at 1 and 4 weeks after surgery (*n* = 5, 2 females and 3 males). (**E**) TEM images of the SSS in sham and JVL rats, the red arrows indicate tight junctions between endothelial cells (scale bars, 1 μm. *n* = 5, 2 females and 3 males). (**F**) Quantification of blood flow in cerebral venous sinuses (CVSs) in sham and JVL rats (*n* = 5, 2 females and 3 males). The data are representative of five independent experiments and are presented as the mean ± SD. *P < 0.05, ***P < 0.001 vs. the respective control by using an unpaired Student’s t test

In cerebral venous circulation, extensive collateral circulation compensation is facilitated by interconnected venous sinuses and the absence of venous valves to compensate for blood flow. Consequently, patients with cerebral venous stenosis often exhibit morphological changes in intracranial veins, which are characterized by abundance, tortuosity, and enlargement. We chose JVL model rats as the experimental group to simulate CVC by ligating the internal jugular veins and posterior glenoid veins [26]. Moreover, sham-operated rats were used as the control group, in which only the skin at the head, neck, and ears was incised with no treatment of the internal jugular veins or posterior glenoid veins. These groups were used to investigate the effects of CVC on the intracranial venous system. Initially, intracranial CE-MRV scans were conducted on sham and JVL rats before the operation (0 days) and at 1 week and 4 weeks postoperation. Compared with those before the operation, the intracranial deep venous system of the rats in the JVL group exhibited marked tortuosity and dilatation at 4 weeks postoperation (Fig. 1A). Additionally, the mean diameters of the SSS and straight sinuses in the JVL rats were notably greater than those in the sham rats (Fig. 1B, C). The pressure within the SSS of the JVL rats was monitored both before and after surgery, and an average pressure of 4.82 mmHg prior surgery, 9.86 mmHg at 1 week after surgery (*P* < 0.001), and 7.25 mmHg at 4 weeks after surgery were observed (*P* < 0.001, Fig. 1D). Furthermore, we analyzed the pathomorphological changes in the endothelial cells of the venous sinus wall in sham and JVL rats via TEM. Compared to those in the sham control group, the vascular endothelial cells in the SSS of the JVL group were moderately edematous, and the number of tight junctions between endothelial cells was reduced (Fig. 1E). Importantly, we measured the blood flow of the cerebral venous sinuses by using laser Doppler flowmetry and detected an ~ 35% decrease in blood flow in JVL rats (Fig. 1F). These observations collectively demonstrate that cerebral venous stasis leads to the CVC phenotypic characteristics, such as tortuous dilation of cerebral venous sinuses, slowing of blood flow, elevated pressure in venous sinuses, and reduction of tight junctions and edema of vascular endothelial cells.

### Transcriptome analysis identified genes linked to platelet activation and blood clotting pathways in the cerebral venous sinuses of JVL rats

**Fig. 2:**
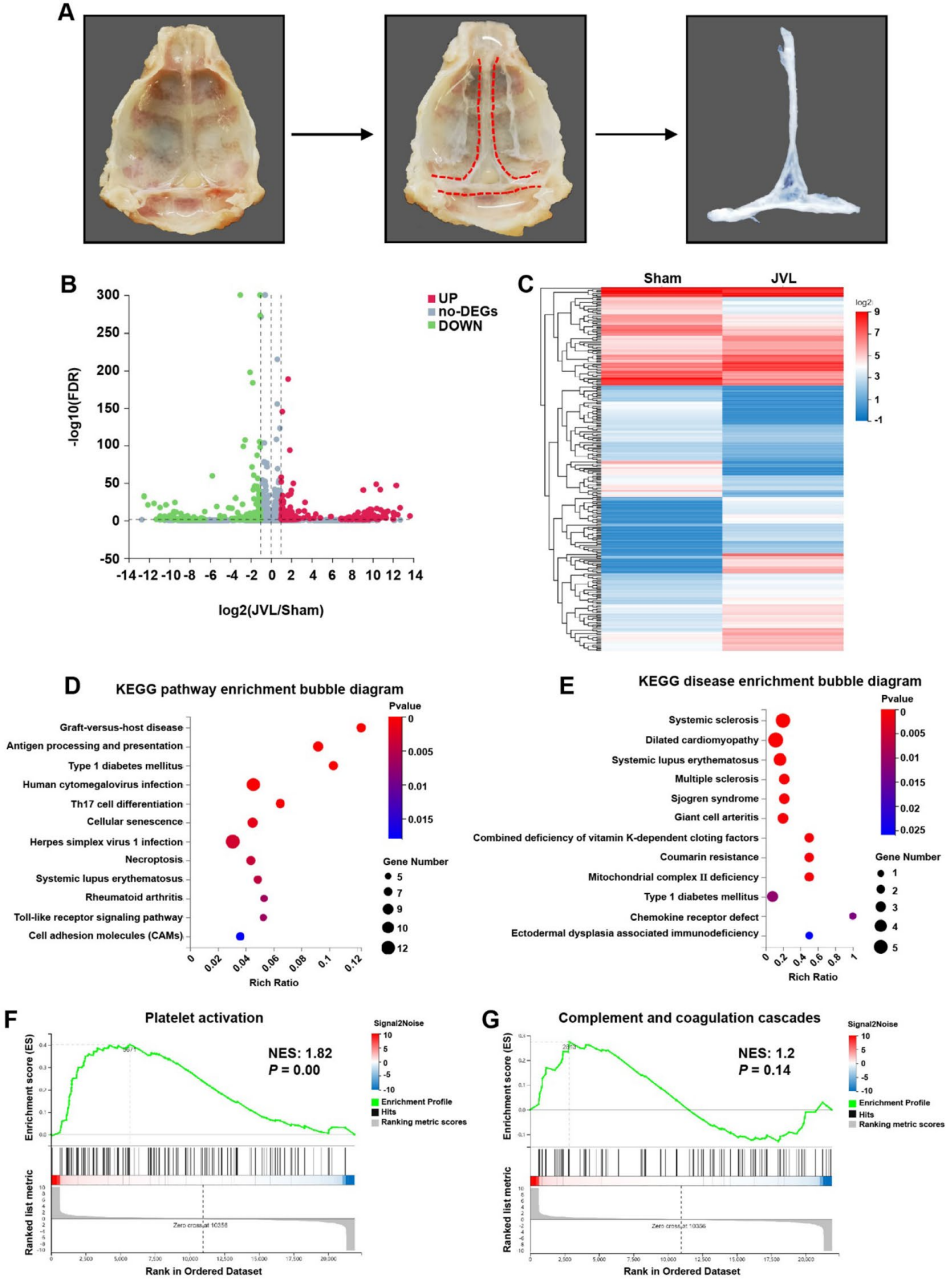
Transcriptome analysis demonstrated the activation of platelet activation and complement and coagulation cascades in endothelial cell injury in the cerebral venous sinus of JVL rats. (**A**) Schematic diagram of cerebral venous sinus isolation. (**B**) Volcano plot of the gene expression profiles. (**C**) Heatmap of DEGs (|log2FC| ≥ 1, *P* ≤ 0.05) in the cerebral venous sinus of sham and JVL rats. (**D**, **E**) Bubble diagram of the top 12 ranked KEGG pathways (**D**) and KEGG diseases (**E**) enrichment analysis of DEGs from the comparison between the sham and JVL group. (**F**, **G**) GSEA plot showing the enrichment of ‘platelet activation’ (**F**, NES: 1.82, *P* = 0.00) and ‘complement and coagulation cascades’ (**G**, NES: 1.2, *P* = 0.14) gene sets in the cerebral venous sinus of JVL rats

To identify novel molecular mechanisms that contribute to endothelial cell damage induced by CVC, the skulls of the rats were surgically excised, and the venous sinuses were isolated from the dura mater with a scalpel under microscopic observation. Subsequently, the venous sinuses were separated from the pristine meninges by using tweezers, and CD45^−^CD31^+^ endothelial cells were isolated from sham (*n* = 6) and JVL (*n* = 6) rats via flow cytometry for transcriptome sequencing analysis (Fig. 2A, Supplemental Fig. 1). We identified 683 DEGs in cerebral venous sinuses between sham and JVL rats, among which 311 were upregulated, and 372 were downregulated (Fig. 2B, C). KEGG pathways (Fig. 2D) and KEGG diseases (Fig. 2E) enrichment analysis showed the top 12 pathways in the bubble diagram, numerous of which were notably enriched, including “Graft-versus-host disease, Antigen processing and presentation, Type 1 diabetes mellitus” among the enriched pathways, and “Systemic sclerosis, Dilated cardiomyopathy, Systemic lupus erythematosus” within the enriched diseases. Interestingly, gene set enrichment analysis (GSEA) demonstrated that the expression of genes involved in platelet activation, complement and coagulation cascades, antigen processing and presentation, endocytosis and the VEGF signaling pathway was significantly upregulated in the cerebral venous sinuses of JVL rats than in those of sham rats (Fig. 2F, G and Supplemental Fig. 2A-C). We further analyzed the expression of genes associated with antigen processing and presentation, endocytosis and the VEGF signaling pathway and demonstrated that almost all of the analyzed genes were expressed in the cerebral venous sinuses of sham rats and strongly upregulated upon cerebral venous stasis (Supplemental Fig. 2D-F). Together, these transcriptomic data indicate that signals associated with JVL (particularly platelet activation and complement and coagulation cascades) may mediate the injury of endothelial cells induced by CVC.

### Impact of cerebral venous congestion on vWF and F8 in complement and coagulation cascades

**Fig. 3:**
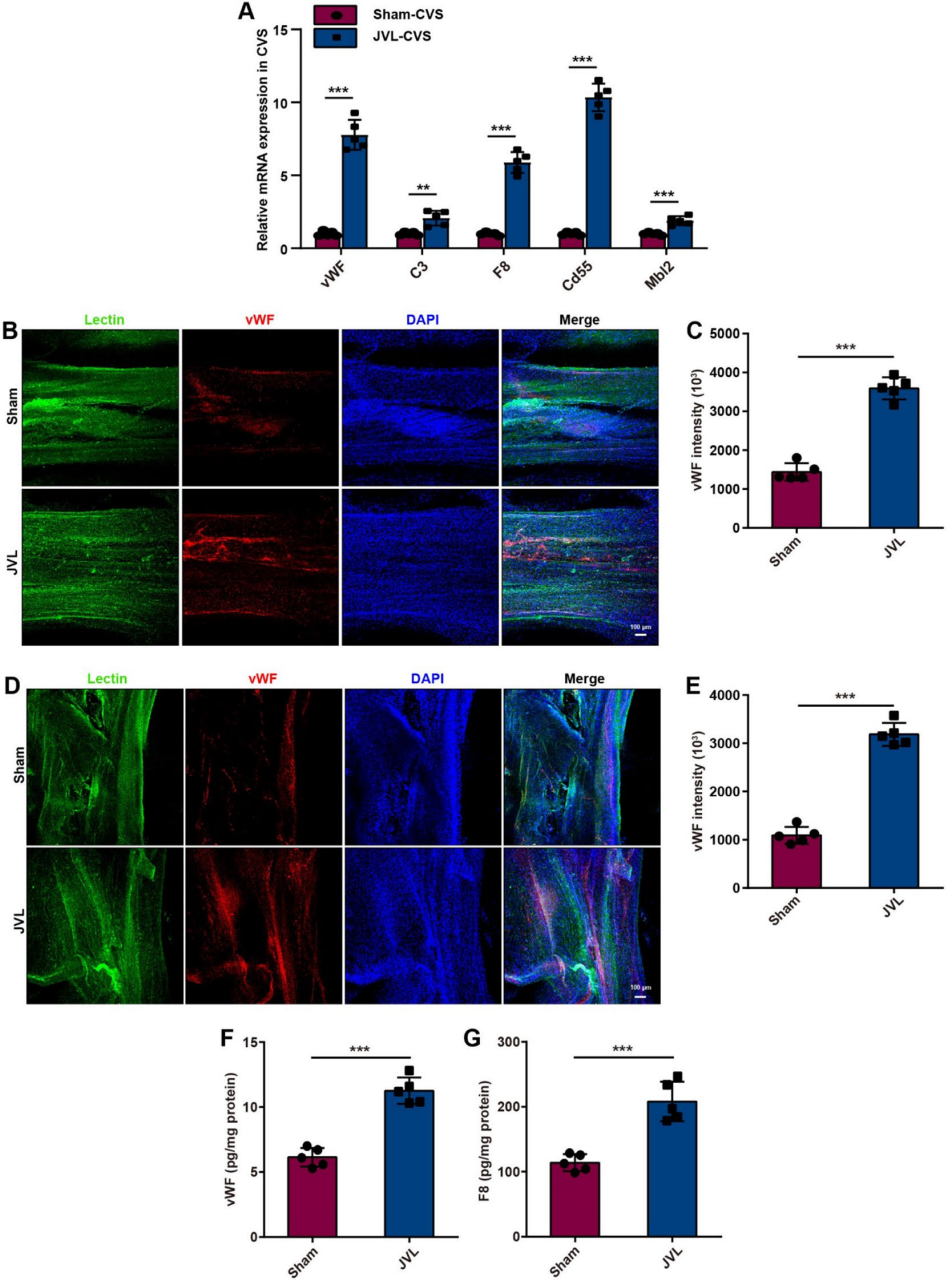
Cerebral venous congestion activates the expression of the clotting factors vWF and F8 in complement and coagulation cascades. (**A**) Relative mRNA expression of vWF, C3, F8, Cd55 and Mbl2 in the cerebral venous sinus (CVS) of sham and JVL rats (*n* = 5, 2 females and 3 males). (**B**, **C**) Representative images and quantification of vWF along the SSS (lectin-green) in sham and JVL rats (scale bars, 100 μm, *n* = 5, 2 females and 3 males). (**D**, **E**) Representative images and quantification of vWF along the TS (lectin-green) in sham and JVL rats (scale bars, 100 μm, *n* = 5, 2 females and 3 males). (**F**, **G**) vWF and F8 levels in the cerebral venous sinus were determined via ELISA and were significantly greater in JVL rats than in sham control rats (*n* = 5, 2 females and 3 males). The data are representative of five independent experiments and are presented as the mean ± SD. **P < 0.01, ***P < 0.001 vs. the respective control by using an unpaired Student’s t test

To further investigate whether and how vascular endothelial injury induced by CVC has a prothrombotic effect, we examined the genes that are involved in the complement and coagulation cascades pathway. Our results indicated that at 4 weeks after JVL, the mRNA expression of vWF, C3, F8, Cd55 and Mbl2 was markedly upregulated in cerebral venous sinuses, as presented in Fig. 3A. Among them, the platelet-adhesive glycoproteins vWF and F8 are key components of the hemostatic system [27, 28]. As expected, immunofluorescence analyses demonstrated that a small amount of vWF was expressed in the lectin-positive SSS and transverse sinus (TS) of sham control rats, whereas vWF expression was significantly upregulated in JVL rats (Fig. 3B-E). Moreover, the cerebral venous sinuses of the JVL rats had greater concentrations of vWF and F8 than those of the sham control rats according to ELISA (Fig. 3F, G). Afterwards, we isolated microvessels from the brain tissue of sham and JVL rats. The intensity of vWF fluorescence in the microvessels of JVL rats increased by more than 60% compared with that in the microvessels of sham rats (Supplemental Fig. 3A, B). Thus, endothelial cells in the cerebral venous sinuses and microvessels of JVL rats showed an increase in vWF and F8 upon cerebral venous stasis. Collectively, these results support the hypothesis that the complement and coagulation cascades are activated during endothelial damage induced by CVC.

### Impact of cerebral venous congestion on fibrinogen gamma chain (Fgg) and coagulation factor II (F2) on platelet activation

**Fig. 4:**
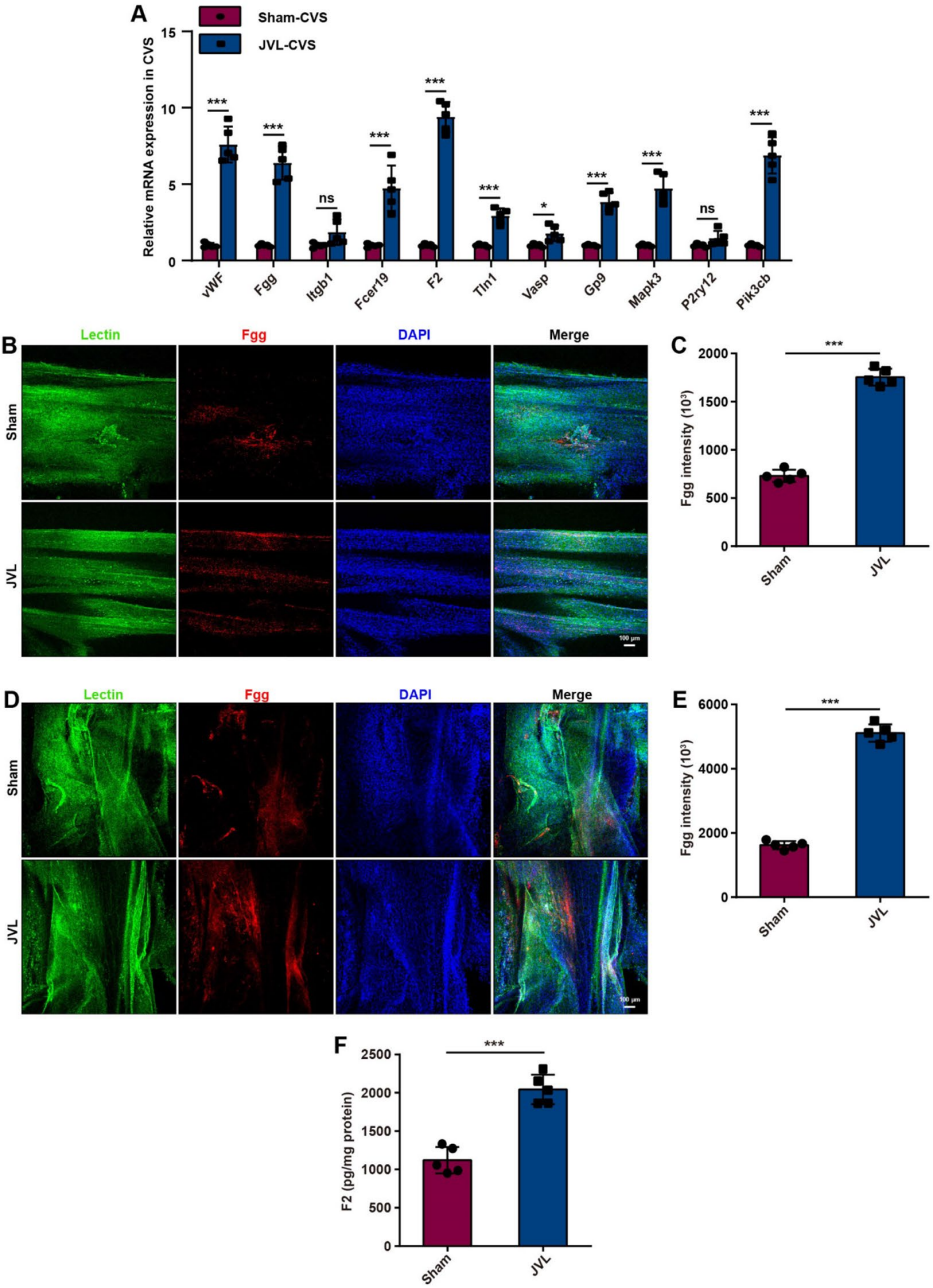
Cerebral venous congestion activates the expression of the clotting factors Fgg and F2 in the platelet activation pathway. (**A**) Relative mRNA expression of vWF, Fgg, Itgb1, Fcer1g, F2, Tln1, Vasp, Gp9, Mapk3, P2ry12 and Pik3cb in the cerebral venous sinus (CVS) of sham and JVL rats (*n* = 5, 2 females and 3 males). (**B**, **C**) Representative images and quantification of Fgg along the SSS (lectin-green) in sham and JVL rats (scale bars, 100 μm, *n* = 5, 2 females and 3 males). (**D**, **E**) Representative images and quantification of Fgg along the TS (lectin-green) in sham and JVL rats (scale bars, 100 μm, *n* = 5, 2 females and 3 males). (**F**) F2 levels in the cerebral venous sinus were determined via ELISA and were significantly greater in JVL rats than in sham control rats (*n* = 5, 2 females and 3 males). The data are representative of five independent experiments and are presented as the mean ± SD. *P < 0.05, ***P < 0.001 vs. the respective control by using an unpaired Student’s t test

We also examined the expression of genes involved in the platelet activation pathway. The results showed that the mRNA expression of vWF, Fgg, Fcer1g, F2, Tln1, Vasp, Gp9, Mapk3 and Pik3cb (but not that of Itgb1 or P2ry12) was notably upregulated in the cerebral venous sinuses of the JVL rats (Fig. 4A). Moreover, the protein levels of Fgg were significantly greater in the SSS, TS and microvessels of JVL rats than in those of sham rats (Fig. 4B-E, Supplemental Fig. 4A, B). ELISA analysis demonstrated that the concentration of F2 in the cerebral venous sinuses of JVL rats was almost two times greater than that in the cerebral venous sinuses of sham control rats (Fig. 4F).

### Cerebral venous congestion induces platelet activation and promotes coagulation

**Fig. 5:**
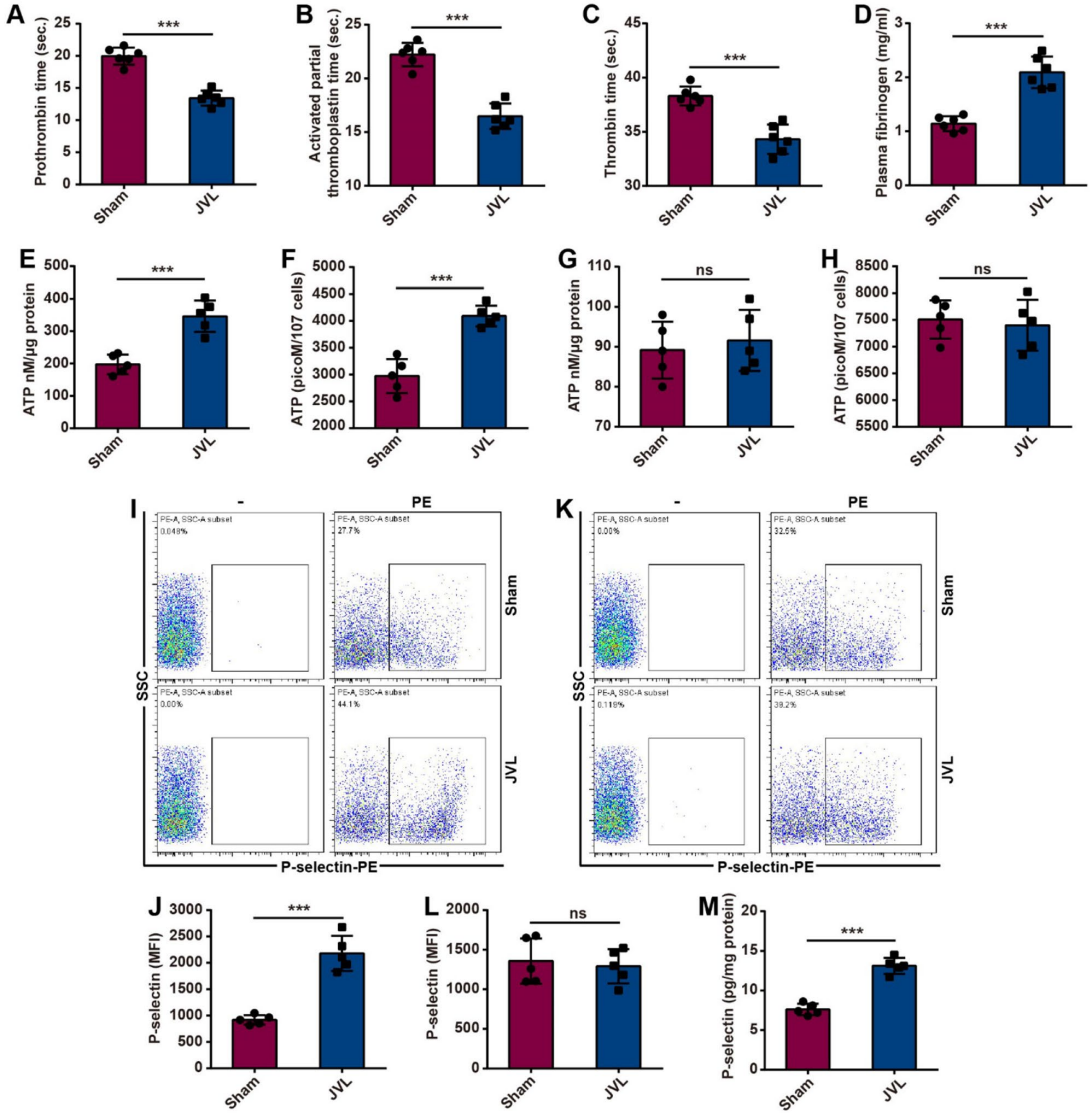
Cerebral venous congestion promotes platelet activation and coagulation. (**A**-**D**) JVL rats had reduced PT (**A**), aPTT (**B**) and TT (**C**) and elevated fibrinogen levels (**D**) (*n* = 5, 2 females and 3 males). (**E**-**H**) Cerebral venous stasis induces ATP release from platelets in the cerebral venous sinus of JVL rats (**E**, **F**) but not in peripheral blood (**G**, **H**) (*n* = 5, 2 females and 3 males). (**I**-**L**) Cerebral venous stasis upregulated platelet P-selectin expression in the cerebral venous sinus of JVL rats (**I**, **J**) but had no effect on platelets in peripheral blood (**K**, **L**) (*n* = 5, 2 females and 3 males). (**M**) There were greater P-selectin concentrations in the cerebral venous sinuses of JVL rats than in those of sham rats (*n* = 5, 2 females and 3 males). The data are representative of five independent experiments and are presented as the mean ± SD. ***P < 0.001 vs. the respective control by using an unpaired Student’s t test

Procoagulant activity during cerebral venous stasis was assessed by measuring coagulation parameters in sham and JVL rats. Compared with those in the sham group, CVC induced by JVL significantly decreased the prothrombin time (PT), activated partial thromboplastin time (aPTT) and thrombin time (TT) of blood in the cerebral venous sinuses and in a hypercoagulable state (Fig. 5A-C). Moreover, the plasma fibrinogen concentration in the cerebral venous sinuses was increased in the JVL rats (Fig. 5D).

Platelets contain two types of organelles: α-granules and dense granules [29]. When activated, platelets release the contents of intracellular granules, such as P-selectin and ATP, and incorporate P-selectin into the plasma membrane, thus resulting in increased P-selectin surface expression on platelets and increased ATP release [30, 31]. We subsequently explored the role of CVC in platelet activation by using platelets from sham and JVL rats. To assess the platelet secretion of dense granules, we measured the release of ATP by using a luciferase-based assay. Platelets from the blood of cerebral venous sinuses in JVL rats showed increased ATP release, whereas no changes in peripheral blood were detected between sham and JVL rats (Fig. 5E-H). To examine platelet exocytosis of α-granules, we measured the externalization of P-selectin via flow cytometry. Compared with those in the blood of sham controls, platelets from the cerebral venous sinuses of JVL rats were found to express P-selectin at significantly greater levels (but not in the peripheral blood) (Fig. 5I-L). ELISA analysis similarly demonstrated that the concentration of P-selectin in the cerebral venous sinuses of JVL rats was greater than that in the cerebral venous sinuses of sham control rats (Fig. 5M). Therefore, endothelial damage caused by CVC hastens coagulation and enhances platelet activation, thereby contributing to hypercoagulable and thrombophilic conditions within the cerebral venous sinuses.

### Upregulation of procoagulant factors in serum from cerebral venous sinuses in patients with internal jugular vein stenosis

**Fig. 6:**
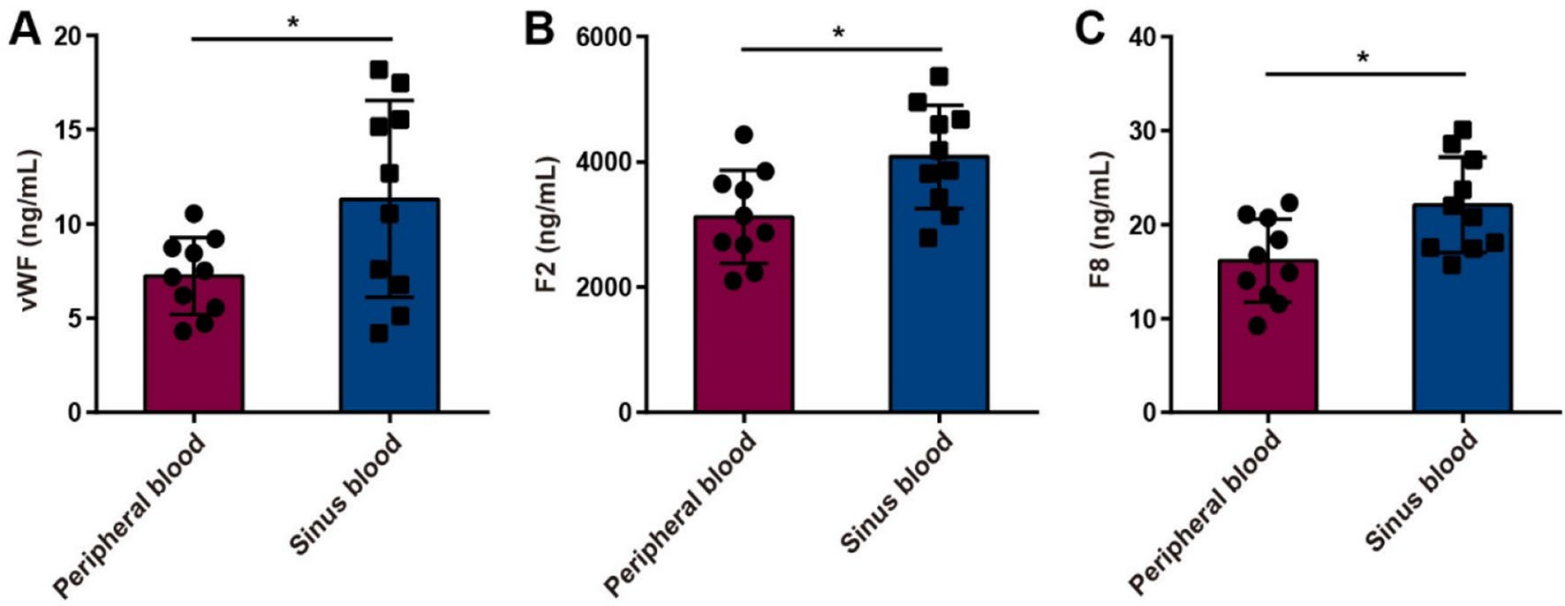
vWF, F2 and F8 concentrations in the peripheral blood and venous sinus blood of internal jugular vein stenosis patients. (**A**-**C**) There were greater vWF (**A**), F2 (**B**) and F8 (**C**) concentrations in the venous sinus blood of internal jugular vein stenosis patients than in the peripheral blood (*n* = 10). The data are representative of ten independent experiments and are presented as the mean ± SD. *P < 0.05 vs. the respective control by using an unpaired Student’s t test

To further verify the effect of cerebral venous stasis on procoagulant factors in patients with CVC caused by jugular vein stenosis, we detected the concentrations of vWF, F2 and F8 in the peripheral blood and venous sinus blood of internal jugular vein stenosis patients by using ELISA. The results showed that the concentrations of vWF, F2 and F8 were greater in the venous sinus blood of internal jugular vein stenosis patients than in the peripheral blood (Fig. 6A-C).

## Discussion

Virchow’s triad theory highlights the risks associated with thrombosis from the impeded circulation of blood. Kim et al. emphasized the hemodynamic consequences of venous sinus occlusion/stenosis, specifically resulting in congestion within the cerebral venous system, which can impede blood flow and heighten the likelihood of thrombosis recurrence within the venous system [32]. The main findings of this study are that CVC intensifies the development of endothelial injury and has a prothrombotic effect in a verified rodent model of cerebral venous occlusion. The obstruction of cerebral venous sinus outflow increases pressure within the cerebral venous sinus, thus leading to vascular endothelial cell damage, which is characterized by impaired tight junctions and reduced antithrombotic function. Although these pathological alterations are commonly observed in cerebral artery stenosis/occlusive disease, their occurrence in conditions related to CVC has received limited attention.

The cerebral venous circulation comprises two components: the deep venous system, which includes the internal cerebral veins, Rosenthal’s vein, and Galen’s vein (as well as their respective side branches), and the superficial venous system, which primarily drains into the dural sinus, SSS, and cavernous sinus. These venous systems ultimately drain into the internal jugular vein through the sigmoid sinus and eventually converge into the right atrium [33–35]. Previous research has demonstrated that the rodent external jugular vein serves as the primary conduit for draining intracranial blood flow, thus excluding venous drainage from the cerebellum. There is extensive anastomosis between the external jugular vein and intracranial veins via the olfactory bulb, medial canthus, and sigmoid sinus, whereas the TS anastomoses with extracranial veins through the posterior glenoid vein [26, 36]. To investigate the potential effects of congestion in the cerebral venous system on thrombosis, we performed animal experiments. Given that the posterior glenoid vein in rodents serves as a direct conduit connecting the TS and the external jugular vein, which is analogous to the J3 segment of the internal jugular vein in humans, we replicated the deceleration or stagnation of blood circulation within the venous sinus by ligating the posterior glenoid vein. Our previous research demonstrated that JVL rats exhibit symptoms of cognitive impairment akin to those observed in individuals with cerebral venous stenosis. Furthermore, metabolomics analysis indicated a reduction in neurotransmitters (particularly GABA) in brain tissue post-JVL, which is consistent with findings in the cerebrospinal fluid of patients with cerebral venous stenosis [26]. This study demonstrated that congestion of blood flow in venous sinuses resulted in changes in the local microenvironment, which contributed to hypercoagulable and thrombophilic conditions. These changes include endothelial cell damage; increased levels of fibronectin; elevated expression of vWF, F8, CD55, and Mb12; and platelet activation within stagnant venous sinuses. These findings provide valuable insights into the pathophysiology of patients with venous thrombosis, especially those with CVT.

Vascular thrombi are generated under conditions of reduced blood flow and shear stress and are mainly composed of fibrin chains, red blood cells and a limited quantity of platelets. Virchow’s triad encompasses three essential components, including intravascular vessel wall injury, impaired hemodynamics and hypercoagulability [37]. An understanding of the factors implicated in the formation of thrombi and subsequent thromboembolic events can help clinicians to categorize risk, guide clinical decision-making pertaining to treatment, and implement preventive measures. In the context of streamlined (laminar) flow, shear stresses on endothelial cells have an impact on their morphology and function. To mitigate these effects, cells elongate and align along the direction of flow. The secretion and release of endothelial defenses such as nitric oxide (NO), prostacyclin (PGI2), and tissue plasminogen activator (t-PA) are contingent on the stresses exerted on the vessel wall [38–41]. Consequently, in the occurrence of endothelial injury, the regulation of blood flow governs vascular reactivity and restricts platelet adhesion, aggregation and fibrin formation solely to the area of endothelial injury. Furthermore, the production and secretion of endothelial mediators with prothrombotic and proinflammatory properties, including tissue factor (TF), vWF, endothelin, ICAM-1, and VCAM-1, are contingent upon the stresses exerted on the vessel wall [42–45]. High shear forces on the vessel wall also trigger platelet activation through the release of vWF, which promotes platelet adhesion to the exposed subendothelium [46]. This study demonstrated that cerebral venous sinus congestion without high shear forces also induces vascular wall damage, thus leading to the upregulation of prothrombotic molecules such as vWF, F8, CD55, and Mb12 in endothelial cells. More importantly, this upregulation also triggers platelet activation within the venous sinus, as evidenced by elevated release of P-selectin and ATP, as well as increased plasma fibrin levels. This pathological observation indicates that venous congestion results in compromised tight junctions and reduced antithrombotic function of endothelial cells, thus elucidating the pathogenesis of venous thrombosis in patients with CVT, patients with chronic heart failure, and astronauts during space travel. Even the implications of this finding on human health are substantial for civilian space travel and forthcoming expeditionary missions, including those to Mars.

However, several limitations of this study should be noted. First, due to the lack of an animal model for thrombus recurrence, this study did not directly observe the formation of thrombi in the venous sinus after ligation of the posterior glenoid vein. Instead, this study solely focused on examining the pathological changes that contribute to hypercoagulable and thrombophilic states. The present study provides valuable empirical evidence for the recognition of Virchow’s triad as follows: endothelial damage or dysfunction (and related structural abnormal changes); abnormal blood stasis; and abnormal hemostasias, platelets, and fibrinolysis. Extensive abnormal changes in these variables are evident in CVC. Thus, CVC could demonstrably drive a prothrombotic or hypercoagulable state by virtue of the fulfillment of Virchow’s triad for thrombogenesis. It is imperative to prioritize research on diseases and conditions associated with CVC, such as spaceflight and chronic heart failure, and venous sinus or vein stenosis/occlusion of the head and neck.

## Conclusion

In summary, our study demonstrates that CVC induces endothelial cell injury as evidenced by increased expression of vWF, Fgg and coagulation factors, as well as platelet activation, which exhibits a procoagulant phenotype and promotes thrombosis. These findings will help to understand the intrinsic mechanisms between Virchow’s Triad in the context of CVC.

## Electronic supplementary material

Below is the link to the electronic supplementary material.

Supplementary Material 1

## Data Availability

The authors confirm that the data supporting the findings of this study are available within the article and its supplementary materials. Additional data are available from the corresponding author upon reasonable request.

## Abbreviations

CVT: Cerebral venous thrombosis
CVC: Cerebral venous congestion
vWF: Von Willebrand factor
JVL: Jugular vein ligation
IJV: Internal jugular vein
PT: Prothrombin time
APTT: Activated partial thromboplastin time
TT: Thrombin time
PRP: Platelet rich plasma
TEM: Transmission electron microscopy
GSEA: Gene set enrichment analysis
TS: Transverse sinus
SSS: Superior sagittal sinus
Fgg: Fibrinogen gamma chain
