# Supplementary material for: Revisiting Virchow’s triad: exploring the cellular and molecular alterations in cerebral venous congestion
Source: Cell Biosci. 2024 Oct 23;14:131. doi: 10.1186/s13578-024-01314-5 (PMC11515517; doi:10.1186/s13578-024-01314-5)
Supplement: Supplementary file 1 — Supplementary Material 1 [file 13578_2024_1314_MOESM1_ESM.docx]

**Supplementary information**

**Revisiting Virchow's Triad: Exploring the Cellular and Molecular Alterations in Cerebral Venous Congestion**

Chen Zhou et al.

Supplementary figures and supplementary tables


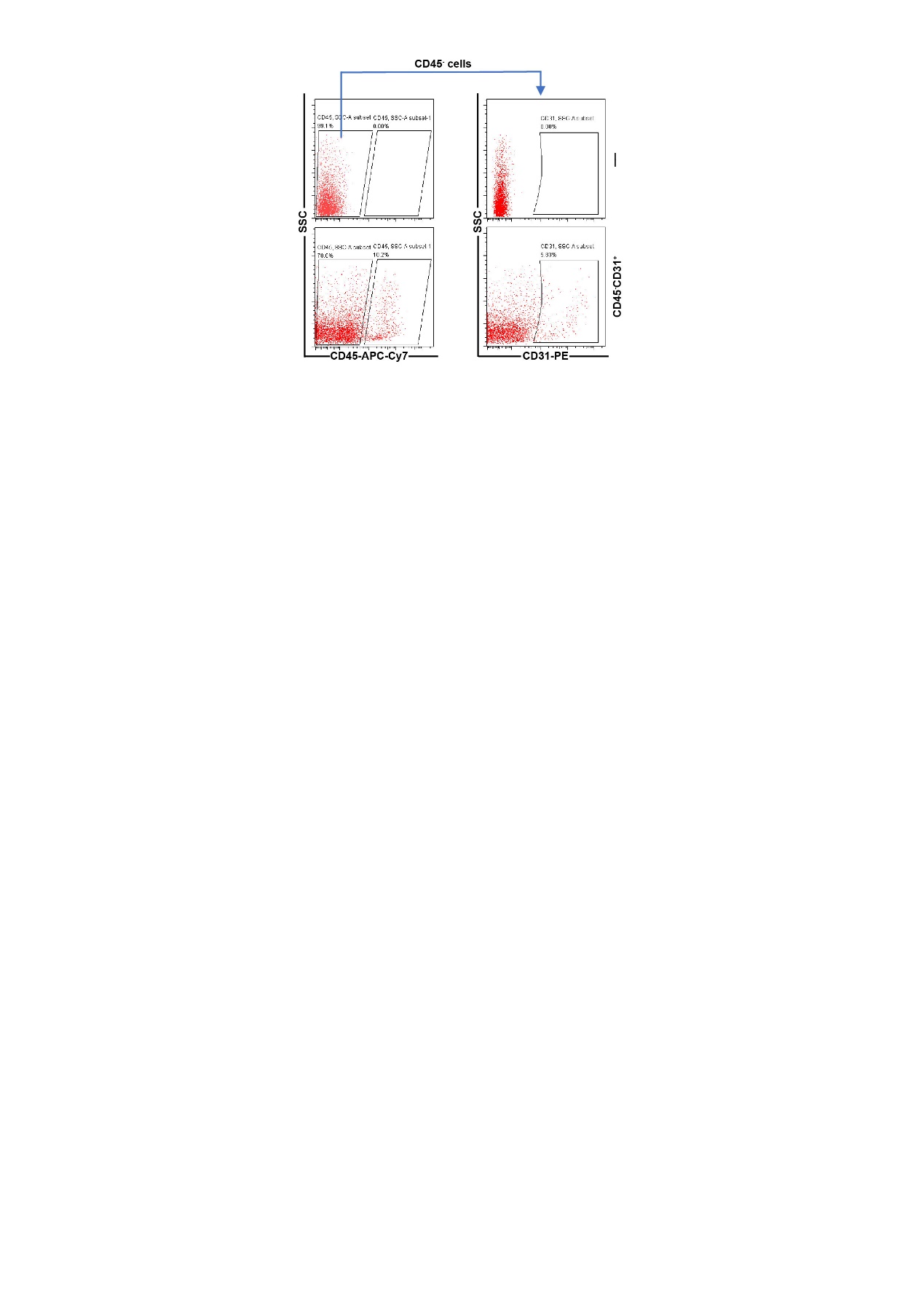


**Supplementary figure 1. The gating and sorting strategy of CD45^-^CD31^+^ endothelial cells of cerebral venous sinuses.**


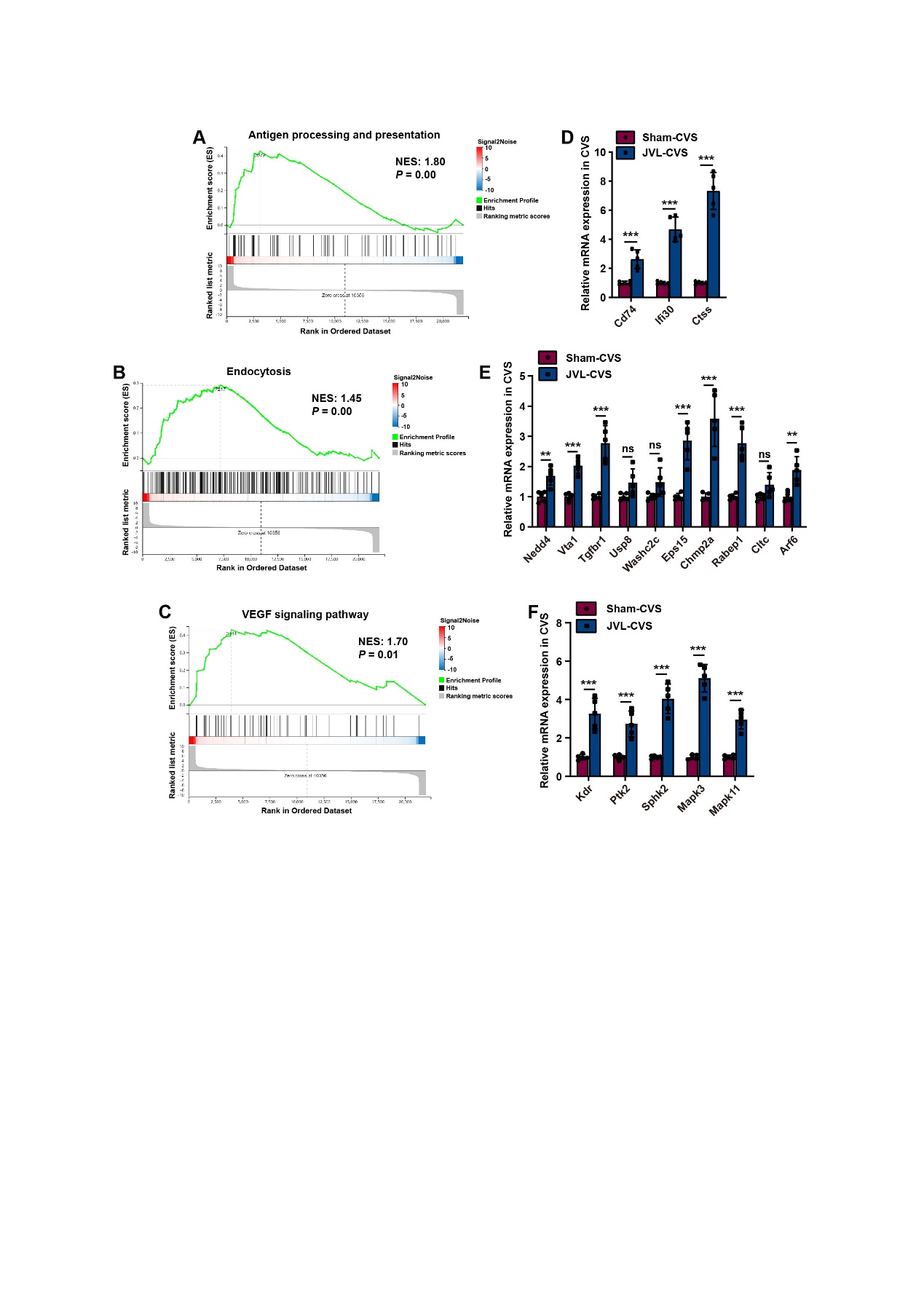


**Supplementary figure 2.** **Transcriptome analysis reveals the activation of Antigen processing and presentation, Endocytosis and VEGF signaling pathway in cerebral venous sinus of JVL rats.** (A-C) GSEA plot showing the enrichment of ‘Antigen processing and presentation’ (NES: 1.80, *P* = 0.00), ‘Endocytosis’ (NES: 1.45, *P* = 0.00) and ‘VEGF signaling pathway’ (NES: 1.70, *P* = 0.01) gene sets in cerebral venous sinus of JVL rats. (D) Relative mRNA expression of Cd74, Ifi30 and Ctss in ‘Antigen processing and presentation’ pathway in cerebral venous sinus of sham and JVL rats (n = 5, 2 females and 3 males). (E) Relative mRNA expression of Nedd4, Vta1, Tgfbr1, Usp8, Washc2c, Eps15, Chmp2a, Rabep1, Cltc and Arf6 in ‘Endocytosis’ pathway in cerebral venous sinus of sham and JVL rats (n = 5, 2 females and 3 males). (F) Relative mRNA expression of Kdr, Ptk2, Sphk2, Mapk3 and Mapk11 in ‘VEGF signaling pathway’ in cerebral venous sinus of sham and JVL rats (n = 5, 2 females and 3 males). The data are representative of five independent experiments and are presented as the mean ± SD. ***P* < 0.01, ****P* < 0.001 vs. the respective control by using an unpaired Student’s t test.


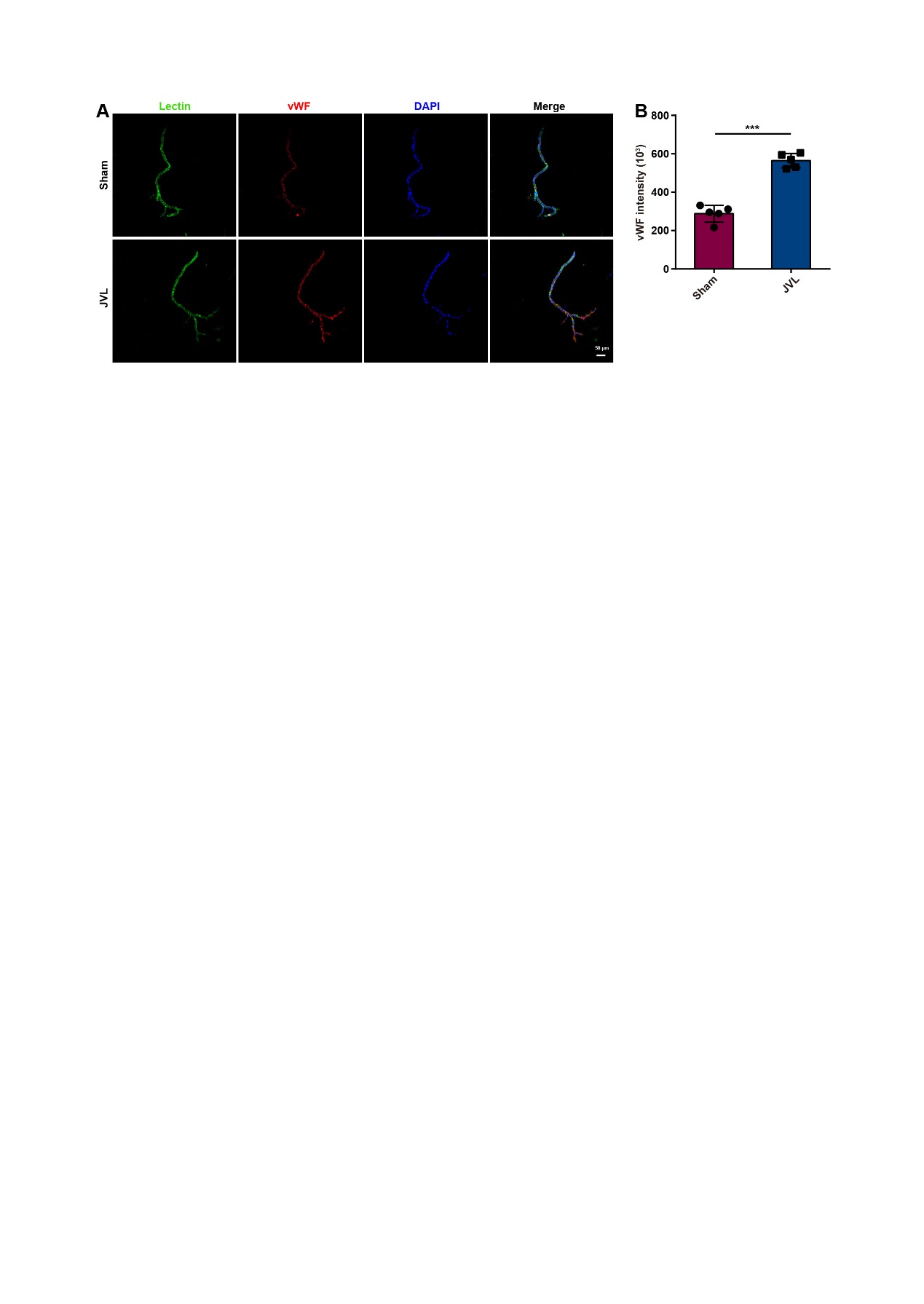


**Supplementary figure 3.** **Cerebral venous congestion promotes the expression of clotting factor vWF in cerebral microvessels of complement and coagulation cascades.** (A, B) Immunofluorescence staining showing the increase of vWF in isolated microvessels labeling with lectin from JVL rats. (Scale bars, 50 μm. n = 5, 2 females and 3 males). The data are representative of five independent experiments and are presented as the mean ± SD. ****P* < 0.001 vs. the respective control by using an unpaired Student’s t test.


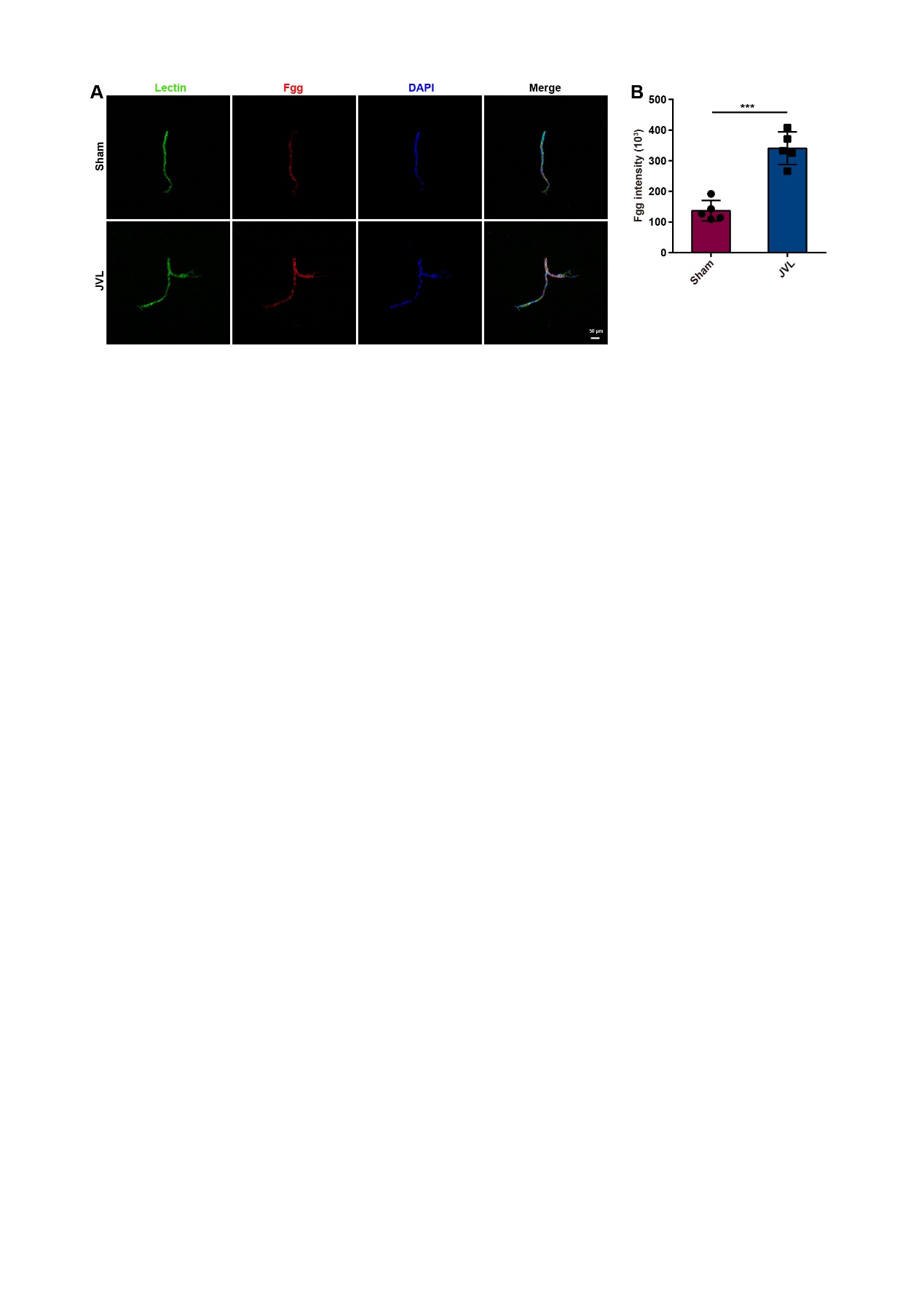


**Supplementary figure 4. Cerebral venous congestion promotes the expression of clotting factor Fgg in cerebral microvessels of platelet activation pathway.** (A, B) Immunofluorescence staining showing the increase of Fgg in isolated microvessels labeling with lectin from JVL rats. (Scale bars, 50 μm. n = 5, 2 females and 3 males). The data are representative of five independent experiments and are presented as the mean ± SD. ****P* < 0.001 vs. the respective control by using an unpaired Student’s t test.

Supplementary Table 1. Primer sequences of oligonucleotides for real-time PCR.

| Rea-Time PCR | |
| --- | --- |
| rat vWF forward | 5’-CCTACGGCTTGCACTATTCA-3’ |
| rat vWF reverse | 5’-GCCAGACACTTGCGTTCATC-3’ |
| rat C3 forward | 5’-ACAAACTGGAGGAACCTTACC-3’ |
| rat C3 reverse | 5’-ACAGGAGGCACAGAGTCAAA-3’ |
| rat F8 forward | 5’-TTGGACCGAGGTTCACGAC-3’ |
| rat F8 reverse | 5’-CCAGAAGGACATACCGACAGC-3’ |
| rat Cd55 forward | 5’-ACGGTGAAGTAGGAGAATGGAG-3’ |
| rat Cd55 reverse | 5’-GGCTTGTGAGACGTTGGTTT-3’ |
| rat Mbl2 forward | 5’-GGCATAACGGACCAGAGGA-3’ |
| rat Mbl2 reverse | 5’-CCCATTTGTCAAGAGCACCAC-3’ |
| rat Fgg forward | 5’-GAAATGGCTGGACCGTATTG-3’ |
| rat Fgg reverse | 5’-CGTATGGGATGGTGGACTGC-3’ |
| rat Itgb1 forward | 5’-AAATGGACGAAAGTGCTCTAACA-3’ |
| rat Itgb1 reverse | 5’-GATGAACTGAAGGACCACCTCTAC-3’ |
| rat Fcer1g forward | 5’-AGCGGTGATCTTGTTCTTGC-3’ |
| rat Fcer1g reverse | 5’-CTGGCTATGTCTGCCTTTCG-3’ |
| rat F2 forward | 5’-AAACCTAGACCGTGACATTGCT-3’ |
| rat F2 reverse | 5’-GCTGGGCTGTATCTCGTTGA-3’ |
| rat Tln1 forward | 5’-ACTATCTGTGCCCGAATTGGTA-3’ |
| rat Tln1 reverse | 5’-TCCCTGTTCCCTCAGTGTCC-3’ |
| rat Vasp forward | 5’-GGGCTACTGTGATGCTTTACG-3’ |
| rat Vasp reverse | 5’-ACTTGACACCTCGAATGATGG-3’ |
| rat Gp9 forward | 5’-GGCTCCTGTTGCTGCTCTG-3’ |
| rat Gp9 reverse | 5’-TGAACGTAGGCTGTTGTTGGT-3’ |
| rat P2ry12 forward | 5’-GATTGATAACCATTGACCGATAC-3’ |
| rat P2ry12 reverse | 5’- GGCAGTGACAGCAGGAACA -3’ |
| rat Pik3cb forward | 5’-CAATCCAGAAACGCCTCACT-3’ |
| rat Pik3cb reverse | 5’-GGCAGCCTCTATGGCAATC-3’ |
| rat Cd74 forward | 5’-CGAAATCTGCCAAACCTGTG-3’ |
| rat Cd74 reverse | 5’-TTGCCATACTTGGTAACATTCTTC-3’ |
| rat Ifi30 forward | 5’-TTGTCGCTACTTCCTCGTCC-3’ |
| rat Ifi30 reverse | 5’-GCCTCCACCTTGTTCAGTTTA-3’ |
| rat Ctss forward | 5’-AGCCATTCCTCCTTCTTCCTCT-3’ |
| rat Ctss reverse | 5’-AAGCCAGTAGTCTTTCCCATCAA-3’ |
| rat Nedd4 forward | 5’-TGAACGACTGGAGGGAACATA-3’ |
| rat Nedd4 reverse | 5’-ATTTGAGCCATAGAGTTCAGCA-3’ |
| rat Vta1 forward | 5’-GACTGTCCCAGCCATTCCC-3’ |
| rat Vta1 reverse | 5’-TACTTCTGAGCCCGAGCAA-3’ |
| rat Tgfbr1 forward | 5’-GATCGCCCTTTCATTTCAGAG-3’ |
| rat Tgfbr1 reverse | 5’-GACCTTTGCCGATGCTTTCT-3’ |
| rat Usp8 forward | 5’-AGAGCTGAGTGCAGGCAAGG-3’ |
| rat Usp8 reverse | 5’-GGGTGAATGGTAGTAACGAAAA-3’ |
| rat Washc2c forward | 5’-AGAAGACTCGGGAACAGAAAGA-3’ |
| rat Washc2c reverse | 5’-GTCAAGTTGCTCAAACGCACTA-3’ |
| rat Eps15 forward | 5’-AAACACCCAGCAAGAGTGAAG-3’ |
| rat Eps15 reverse | 5’-ATTGGCAGTGGTAGAAGAAGAA-3’ |
| rat Chmp2a forward | 5’-CGGAAGACGCCAGAGGAAC-3’ |
| rat Chmp2a reverse | 5’-CGCACATATCGCCGGGTA-3’ |
| rat Rabep1 forward | 5’-CTGAGGCTGGAGCAGGAAC-3’ |
| rat Rabep1 reverse | 5’-CATCACGACAGAGCGAAGC-3’ |
| rat Cltc forward | 5’-CTGTGACCGCTTTGACTTCG-3’ |
| rat Cltc reverse | 5’-CAACATCCAGCAATCCTCCA-3’ |
| rat Arf6 forward | 5’-CTTCGGGAACAAGGAAATGC-3’ |
| rat Arf6 reverse | 5’-TGTAGTAATGCCGCCAGAGC-3’ |
| rat Kdr forward | 5’-GAAATTACTGTCCAGCCTGCTA-3’ |
| rat Kdr reverse | 5’-GACCGATGTTGCCTGTGAG-3’ |
| rat Ptk2 forward | 5’-AGCAGTAATGAGCCAACCAC-3’ |
| rat Ptk2 reverse | 5’-TGAGGCGAAATCCATAGCAG-3’ |
| rat Sphk2 forward | 5’-TGGGCTGTCCTTCAACCTC-3’ |
| rat Sphk2 reverse | 5’-GCTAGTGCATTGCCCGAAC-3’ |
| rat Mapk3 forward | 5’-CCAGAGTGGCTATCAAGAAGA-3’ |
| rat Mapk3 reverse | 5’-CTCCATGAGGTCCTGAACAAT-3’ |
| rat Mapk11 forward | 5’-CCTACGGCTCGGTCTGTTC-3’ |
| rat Mapk11 reverse | 5’-CCTATGACGTTCTCGTGCTT-3’ |

Supplementary Table 2. List of antibodies with their sources and experimental conditions.

| Protein | Species | Application | Manufacturer | Catalog No. | Dilution |
| --- | --- | --- | --- | --- | --- |
| anti-vWF | Rabbit | IF | Proteintech | 27186-1-AP | 1:100 |
| anti-Fgg | Rabbit | IF | Proteintech | 15841-1-AP | 1:100 |
| anti-CD31-PE | Mouse | FC | BD | 555027 | 1:100 |
| P-selectin-PE | Mouse | FC | Biolegend | 148305 | 1:100 |
| AlexaFluor^®^ 594-conjugated Goat anti-rabbit IgG | Goat | IF | Abcam | ab150160 | 1:200 |

IF: Immunofluorescence; FC: Flow cytometry
